# Assessing methanotrophy and carbon fixation for biofuel production by* Methanosarcina acetivorans*

**DOI:** 10.1186/s12934-015-0404-4

**Published:** 2016-01-17

**Authors:** Hadi Nazem-Bokaee, Saratram Gopalakrishnan, James G. Ferry, Thomas K. Wood, Costas D. Maranas

**Affiliations:** Department of Chemical Engineering, The Pennsylvania State University, University Park, PA 16802 USA; Department of Biochemistry and Molecular Biology, The Pennsylvania State University, University Park, PA 16802 USA

**Keywords:** *Methanosarcina acetivorans*, Genome-scale metabolic model, Methane utilization

## Abstract

**Background:**

*Methanosarcina acetivorans* is a model archaeon with renewed interest due to its unique reversible methane production pathways. However, the mechanism and relevant pathways implicated in (co)utilizing novel carbon substrates in this organism are still not fully understood. This paper provides a comprehensive inventory of thermodynamically feasible routes for anaerobic methane oxidation, co-reactant utilization, and maximum carbon yields of major biofuel candidates by *M. acetivorans*.

**Results:**

Here, an updated genome-scale metabolic model of *M. acetivorans* is introduced (iMAC868 containing 868 genes, 845 reactions, and 718 metabolites) by integrating information from two previously reconstructed metabolic models (i.e., iVS941 and iMB745), modifying 17 reactions, adding 24 new reactions, and revising 64 gene-protein-reaction associations based on newly available information. The new model establishes improved predictions of growth yields on native substrates and is capable of correctly predicting the knockout outcomes for 27 out of 28 gene deletion mutants. By tracing a bifurcated electron flow mechanism, the iMAC868 model predicts thermodynamically feasible (co)utilization pathway of methane and bicarbonate using various terminal electron acceptors through the reversal of the aceticlastic pathway.

**Conclusions:**

This effort paves the way in informing the search for thermodynamically feasible ways of (co)utilizing novel carbon substrates in the domain *Archaea*.

**Electronic supplementary material:**

The online version of this article (doi:10.1186/s12934-015-0404-4) contains supplementary material, which is available to authorized users.

## Background

Methane, the second most important greenhouse gas, is regulated primarily by microbial processes [1]. A renewed interest in methane as a gas substrate for the production of biofuels is spearheaded by its abundance in shale gas [2–5]. At the same time, concerns related with methane’s role as a potent greenhouse gas drives the need to mitigate its adverse environmental impact [6]. Advances in the characterization of microbial consortia in anoxic sediments have revealed the potential of transforming methane into various products through biological routes [7–10].

The global methane cycle is predominantly controlled by anaerobic methanotrophic archaea (ANME) in anoxic environments [11, 12] and aerobic methanotrophic bacteria at the anoxic–oxic interface of habitats [13, 14]. Aerobic methanotrophy [15], proceeds via the oxidation of methane to methanol by a methane monooxygenase and then to formaldehyde by methanol dehydrogenase, which is subsequently integrated into central carbon metabolism through the ribulose monophosphate or the serine pathway [16]. This scheme, however, requires an initial activation cost in the form of NAD(P)H, which is replenished at the expense of carbon efficiency. Shaped by the paucity of available energy, the anaerobic methanotrophy has been shown to exhibit better carbon and energy efficiency [17, 18]. However, in contrast to the aerobic route, anaerobic methanotrophy is relatively poorly characterized as a result of the difficulties in culturing ANMEs in the lab [11] arising from syntrophy requirements. In such environments, the anaerobic methanotroph oxidizes methane and the microbial partner reduces an electron acceptor, often an inorganic ion such as NO_3_^−^ [19] or SO_4_^2−^ [20]. Despite these difficulties, recent metagenomics analysis of ANMEs has partially revealed the methanotrophic pathways, observed in most methanogenic archaea, demonstrating the phylogenetic relationship between ANMEs and methanogens [21, 22]. Of particular interest is the methanogenic archaeon *Methanosarcina acetivorans* for which trace methane oxidation has been observed [23, 24] implying that it possesses the necessary pathways and electron flow systems to accomplish methanotrophy. However, a complete reversal of methanogenesis pathway is thermodynamically infeasible unless coupled with an electron-accepting pathway [25, 26].

*M. acetivorans*, a strictly anaerobic marine methanogen possessing one of the largest known archaeal genomes [27], has emerged as a model archaeon owing to the availability of genetic tools [28] and versatility in substrate utilization [29, 30]. While the pathways describing the metabolism on native substrates have been extensively studied [31–34], relevant pathways and electron flows for methane oxidation by *M. acetivorans* remain largely uncharacterized. Two genome-scale metabolic (GSM) models, iVS941 [35] and iMB745 [36], for this organism have been proposed. Both models, however, are not up to date with the current literature on the stoichiometry of ion transport across the membrane and ATP synthesis [37–42]. Recent findings on the electron flow mechanisms of *M. acetivorans* cell extracts grown with methane (unpublished observations, Zhen Yan and James G. Ferry) motivates an update in the existing genome-scale models to incorporate recent findings and to allow for the analysis of methane utilization in silico.

In this paper we make use of a revamped GSM for *M. acetivorans* to postulate pathways for reversing methanogenesis while maintaining overall thermodynamic feasibility. We first generated an up-to-date GSM model for *M. acetivorans* by combining information from two earlier models (i.e., iVS941 and iMB745) along with the most recent data from literature and databases. *M. acetivorans* has transcriptome and proteome profiles that differ depending on growth substrate [31, 34]. We augmented the updated gene-protein-reaction (GPR) associations with regulatory (i.e., −R) switches to incorporate proteomics data to the updated metabolic reconstruction by switching on/off reactions for different substrates. Using the model as a starting point a thermodynamically feasible pathway is proposed for the co-utilization of methane and bicarbonate in the presence of Fe^3+^, NO_3_^−^, SO_4_^2−^, and MnO_2_ as external electron acceptors. Overall ΔG ≤ 0 is imposed as a constraint to ensure thermodynamic feasibility of methanogenesis reversal in the presence of an external electron acceptor. The interplay between externally supplied electron acceptors and various by-products is analyzed. The feasibility of methanotrophy by resting cells is assessed when all carbons coming from methane and bicarbonate are converted into acetate, formate, CO_2_, and methyl sulfide, the known byproducts of *M. acetivorans*’ metabolism [30, 43] some of which were also observed recently by Wood et al. [44] as end products of methanotrophy by the archaeon.

## Results and discussion

### Updated genome-scale metabolic model reconstruction of *M. acetivorans*, iMAC868

iMAC868 contains 868 genes, 845 reactions, and 718 metabolites (Additional file 1) and provides better agreement with the observed growth yields on methanol and acetate compared to earlier reconstructions (see Table 1). Improved prediction is due to the correction of charge and mass imbalances of the reactions inherited from the previous metabolic models, incorporation of accurate ion exchange stoichiometries for membrane-bound reactions, and optimization of Na^+^/H^+^ ratio for sodium/proton antiporter (Mrp) and ATP synthase. Among the charge re-balanced reactions, those involving cofactor F_420_ in the methylotrophic pathway and ATP synthase also required proton rebalancing to accurately account for proton exchange across the cell membrane. The number of Na^+^ pumped out by ferredoxin-dependent methanophenazine reductase (Rnf) was updated from three Na^+^ in iMB745 [36] to four Na^+^ per methanophenazine reduced in accordance with experimental findings [38]. In addition to this, the ATP synthase reaction was modified to co-utilize Na^+^ and H^+^ gradients [37]. Using the procedure described in Methods section, the optimal Na^+^/H^+^ ratio recapitulating the growth yields on native substrates was estimated to be 2:1 for Mrp and 3:1 and 1:2 for ATP synthase, respectively. The two identified solutions for ATP synthase are rendered equivalent by the reversible 2:1 antiport of Na^+^/H^+^ across the cell membrane by Mrp, which makes one intracellular H^+^ equivalent to two extracellular Na^+^. We also added an F_420_-dependent NADP reductase to the iMAC868 model (personal communications with James G. Ferry), which functions as a source of NADPH for cell biosynthesis.

**Table 1:** Growth yield predictions of iMAC868 model of *M. acetivorans* compared with predictions of previous models iVS941 [35] and iMB745 [36]

Substrate	Observed growth yield	Predicted growth yield		
		iVS941 [35]	iMB745 [36]	iMAC868^[this study]^
Methanol	5.2 [69]	9.5	4.0	5.26
Acetate	2.4 [29]	4.0	3.0	2.6

Yield units are gram dry cell weight per mol of substrate

Upon correcting 64 GPRs based on updated gene annotations (Additional file 2) and implementing proteomics-dependent growth condition-specific R-GPR switches, iMAC868 correctly predicts gene knockout outcomes for 27 out of 28 mutants of *M. acetivorans* (see Table 2; Additional file 1). The only false prediction by the iMAC868 model is missing the in vivo essentiality of the mutant lacking methanol-specific methyltransferases (Δ*mtaA1* Δ*mtaCB1* Δ*mtaCB2* Δ*mtaCB3*) growing with acetate due to the unknown role of the enzymes in acetate-grown cells [45]. The model correctly captures the essentiality of *mch* [46] by identifying the role of the methylotrophic pathway as a source of reduced F_420_ for NADPH production in acetate-grown cells thereby rendering *mch* (methenyl-H_4_SPT cyclohydrolase) essential. Comparing with the iVS941 model, the iMAC868 model correctly predicts the essentiality of *rnf*, *mtr*, and the membrane-bound *hdr* due to the updated GPRs and ion transport stoichiometries included in this model.

**Table 2:** Gene deletion lethality predictions by iMAC868 model of *M. acetivorans* compared with predictions of previous models

Gene deletion	Acetate			Methanol			References
	iVS941	iMB745	iMAC868	iVS941	iMB745	iMAC868	
*ΔackΔpta*	NGNG	NGNG	NGNG	GG	GG	GG	[30]
*ΔhdrABC*	GG	GG	GG	GG	GG	GG	[70]
*ΔhdrED*	GNG	NGNG	NGNG	GNG	NGNG	NGNG	[70]
*Δmch*	GNG	GNG	NGNG	NGNG	NGNG	NGNG	[46]
*ΔmtaA1*	GG	GG	GG	NGNG	NGNG	NGNG	[45]
*ΔmtaB 1C1ΔmtaB 2C2ΔmtaB 3C3*	GG	GG	GG	NGNG	NGNG	NGNG	[45]
*ΔmtaA1ΔmtaB 1C1ΔmtaB 2C2ΔmtaB 3C3*	GNG	GNG	GNG	NGNG	NGNG	NGNG	[45]
*ΔmtbA*	GG	GG	GG	GG	GG	GG	[45]
*ΔmtsDΔmtsFΔmtsH*	GG	GG	GG	GG	GG	GG	[71]
*ΔmtsXΔmtsY, X and Y any two mts genes*	GG	GG	GG	GG	GG	GG	[71]
*ΔrnfHCDGEABF*	GNG	NGNG	NGNG	GG	GG	GG	[70]
*ΔlysK*	GG	GG	GG	GG	GG	GG	[72]
*ΔlysS*	GG	GG	GG	GG	GG	GG	[72]
*Δmtr*	GNG	NGNG	NGNG	NGNG	NGNG	NGNG	[73]
Total correct	9/14	12/14	13/14	13/14	14/14	14/14	

*GG* growth in silico/growth in vivo, *GNG* growth in silico/no growth in vivo, *NGNG* no growth in silico/no growth in vivo

### Model customization to capture methanotrophy by *M. acetivorans*

In order to allow for methanotrophy, the iMAC868 model was customized to enable three new processes: (1) reversal of methyl-coenzyme M reductase (Mcr) reaction, (2) inclusion of a cytosolic methyltransferase (CmtA), and (3) inclusion of a mechanism enabling electron bifurcation and its subsequent discharge to an external electron acceptor. The methyl-coenzyme M reductase of an anaerobic methanotroph (ANME-MCR), capable of oxidizing methane [47], was appended to the iMAC868 model upon deactivating the native Mcr to prevent methanogenesis based on evidence regarding the reversal of methanogenesis in *M. acetivorans* [23, 24, 48], reversibility of native Mcr [49], and the confirmed heterologous expression of ANME-MCR in *M. acetivorans* demonstrating ferric-dependent methanotrophy [44]. CmtA [50] serves as a soluble alternative to membrane-bound Mtr, allowing the conversion of methyl-coenzyme M to methyl-tetrahydrosarcinapterin without drawing on sodium ion gradients across the membrane. Cdh, a key enzyme in the modeled pathway, is dependent on ferredoxin to reduce CO_2_ that generates the carbonyl group in acetyl-CoA [51]. Two flavin-based mechanism are postulated in which an electron pair derived from oxidation of coenzyme B and coenzyme M (*E*_m_ = −143 mV) bifurcates yielding a high-potential electron reducing Fe^3+^ to Fe^2+^ (*E*_m_ = +770 mV) and a low-potential electron reducing ferredoxin (*E*_m_ = −420 mV). Flavin-based electron bifurcation is common among anaerobic microbes including methanogens [52, 53]. Although, both postulated mechanisms depend on delivering electrons to Fe^3+^ on the outer aspect of the cytoplasmic membrane, the bifurcation event occurs either at the cytoplasm or the membrane. Bifurcation in the membrane depends on the Rnf complex, abundant in acetate-grown *M. acetivorans*, which interacts with ferredoxin and contains two FMN-bound subunits that are possible sites for electron bifurcation [54]. Oxidation of coenzyme B and coenzyme M is catalyzed by the membrane-bound CoMS-SCoB heterodisulfide reductase (HdrDE) [54]. Methanophenazine (MP) is a quinone-like electron carrier that shuttles electrons between HdrDE and the Rnf complex. Importantly, the reduction of ferredoxin is not dependent on a sodium gradient. Bifurcation in the cytoplasm is postulated to be dependent on the fused HdrA2:MvhD protein shown previously to be present in acetate-grown *M. acetivorans* [34]. Oxidation of HS-CoB and HS-CoM is catalyzed by the soluble heterodisulfide reductase (HdrB2) that donates electrons to the flavin-containing HdrA2 component where bifurcation takes place reducing ferredoxin and transferring an electron to the membrane where reduction of Fe^3+^ takes place. Finally, an electron transfer reaction is included in the model to transfer the electrons from reduced methanophenazine to an externally supplied electron acceptor based on its reported in vivo essentiality [19, 20, 55]. The essentiality of this reaction was confirmed by the absence of any in silico external electron acceptor-independent thermodynamically feasible metabolic state despite allowing the production of all reported reduced products such as hydrogen gas [56] and organic acids such as acetate and formate [30]. These additions complete the pathways for the oxidation of methane to various end products such as acetate, formate and CO_2_. The addition of prospective biofuel molecule production pathways for ethanol [57], butanol [58], and isobutanol [59] to the model allows the exploration of their thermodynamically feasible maximum theoretical yields for different electron acceptors.

### Products of electron-acceptor-dependent AOM

**Fig. 1:**
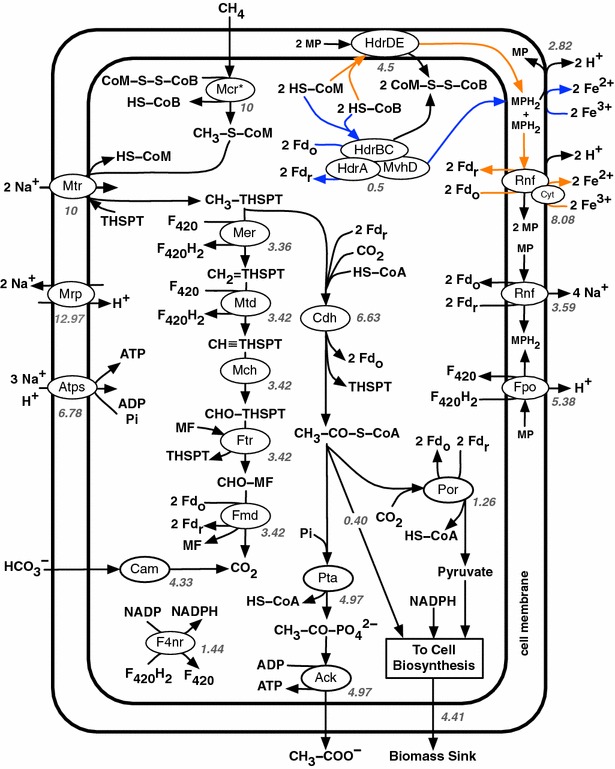
Proposed methanogenesis reversal pathway supported by the iMAC868 model of *M. acetivorans* for co-metabolization of methane and bicarbonate in the presence of Fe^3+^ as external electron acceptor. Soluble and membrane-bound electron bifurcation routes are shown as *blue* and *orange*, respectively, and enzymes within ovals. In both routes, electrons originate from coenzyme B and coenzyme M. For enzymes with multiple subunits, only the subunits of soluble Hdr and Rnf involved in electron bifurcation are shown in detail. *Numbers in italics* next to enzyme ovals denote reaction fluxes (in mmol/gDCW-h) calculated under maximization of acetate production at bicarbonate to methane ratio of 0.44. This ratio corresponds to the maximum thermodynamically feasible value ensuring biomass production at 30 % of its theoretical maximum for Fe^3+^ as the electron acceptor. The flux towards growth was calculated by assuming that 1 g of biomass contains 36 mmol of carbon. Intracellular proton and water stoichiometries are omitted for the sake of simplicity. Soluble methyltransferase (CmtA) is not present in the network since the minimum possible flux through this reaction is zero. *Mcr* ^*^ putative ANME-like Mcr homolog to methyl-coenzyme M reductase, *HdrBC:HdrA:MvhD* soluble ferredoxin-dependent heterodisulfide reductase, *Mtr* methyl-THSPT:coenzyme M methyltransferase, *Mer* methenyl-THSPT reductase, *Mtd* methenyl-THSPT dehydrogenase, *Mch* methenyl-THSPT cyclohydrolase, *Ftr* formylmethanofuran:THSPT formyltransferase, *Fmd* formylmethylfuran dehydrogenase, *Cdh* CO dehydrogenase, *Pta* phosphotransacetylase, *Ack* acetate kinase, *Por* pyruvate synthase, *Atps* ATP synthase, *Mrp* sodium/proton antiporter, *Rnf* methanophenazine reductase, *Cyt* cytochrome c subunit of Rnf complex, *Fpo* F_420_ dehydrogenase, *Cam* carbonic anhydrase, *F4nr* F_420_-dependent NADP reductase, *THSPT* tetrahydrosarcinapterin, *MF* methanofuran, *MP* methanophenazine, *MPH*_*2*_ reduced methanophenazine, *Fd*_*o*_ oxidized ferredoxin, *Fd*_*r*_ reduced ferredoxin, *F*_*420*_ coenzyme F_420_, *F*_*420*_ *H*_*2*_ reduced coenzyme F_420_

The model supports acetate, CO_2_ and biomass as the main products of methanotrophy using all tested electron acceptors. Methane is oxidized to methyl-H_4_SPT by ANME-MCR and Mtr (or CmtA), a part of which is oxidized via the methylotrophic pathway to produce intracellular CO_2_. The remaining methyl-H_4_SPT is used to produce acetyl-CoA, the primary building block for all biomass precursors. Additional carbon fixation occurs via reductive carboxylation by Cdh and Por. Acetyl-CoA is also converted to acetate, generating ATP via substrate-level phosphorylation (Fig. 1). The electrons released upon activation of methane by ANME-MCR are transferred to ferredoxin via soluble and membrane-bound electron transport chains involving flavin-based electron bifurcation mechanisms. In addition, further oxidation of methyl-H_4_SPT through methylotrophic pathway generates reducing equivalents in the form of ferredoxin and F_420_. Fpo and Rnf complexes facilitate the transfer of electrons from reduced F_420_ and ferredoxin, respectively, to the external electron acceptor via methanophenazine, thereby generating H^+^ and Na^+^ gradients across the membrane for chemiosmotic ATP synthesis (see Fig. 1). The primary carbon fixation mechanism via reductive carboxylation prompted a quantitative analysis of the impact of utilizing CO_2_ as a co-substrate in the form of bicarbonate on acetate and biomass yields.

**Fig. 2:**
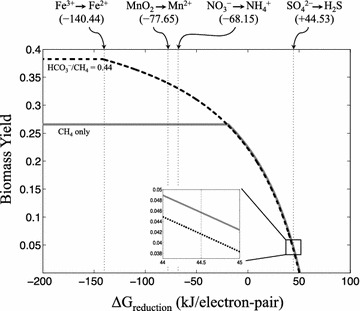
Biomass yield (per 10 mmol methane) as a function of the ΔG of external electron acceptor reduction (kJ/electron-pair) predicted by the iMAC868 model of *M. acetivorans*. *Solid line* methane as the sole carbon source; *dashed line* bicarbonate and methane (at a ratio of HCO_3_^−^/CH_4_ = 0.44) as carbon sources. *Vertical dotted lines* show the ΔG (kJ/electron-pair) of reduction for Fe^3+^ (−140.44), MnO_2_ (−77.65), NO_3_^−^ (−68.15), and SO_4_^2−^ (+44.53). A *magnified insert* shows the maximum biomass yields for sulfate-dependent methanotrophy. All ΔG values were evaluated at pH of 7, 25 °C, and an ionic strength of 0.25 M as described by Alberty [64]

Thermodynamic feasibility of methanotrophy is ensured only when the free energy of reduction (ΔG_red_) of the supplied electron acceptor is less than 50.5 kJ/electron-pair (Fig. 2), corresponding to the maximum free energy equivalents generated by CO_2_ production (see Table 3). Using methane as the sole carbon source, maximum biomass yield is constrained by thermodynamic feasibility when ΔG_red_ of the electron acceptor is greater than −20 kJ/electron-pair. Sulfate-dependent methanotrophy falls within this regime, in which thermodynamic coupling with an exergonic pathway such as acetate or CO_2_ production (Table 3) drives only partial conversion of methane to biomass. In contrast, biomass production is limited only by stoichiometry during ferric-dependent methanotrophy due to the far greater free energy equivalents produced by the reduction of Fe^3+^ to Fe^2+^ (ΔG = −140.44 kJ/electron-pair) compared to SO_4_^2−^ reduction (ΔG = 44.53 kJ/electron-pair). This thermodynamic advantage for Fe^3+^ allows for the co-utilization of bicarbonate up to a maximum HCO_3_^−^/CH_4_ ratio of 0.44 with complete incorporation of all substrate carbons into biomass. However, the endergonic nature of bicarbonate uptake disallows HCO_3_^−^/CH_4_ co-utilization for increasing biomass yield under sulfate-dependent methanotrophy. Methanotrophy using NO_3_^−^ and MnO_2_ also allows for co-utilization of bicarbonate as both electron acceptors have ΔG_red_ greater than −20 kJ/electron-pair (see Table 3).

**Table 3:** Oxidation half reactions of methane to various products and reduction half reactions of various electron acceptors

Oxidation half reactions	ΔG (kJ/mole product)	ΔG (kJ/mole methane)
$CH_{4} + 2 H_{2} O \to CO_{2} + 8 H^{ + } + 8 e^{ - }$	−202.79	−202.79
$2 CH_{4} + 2 H_{2} O \to CH_{3} COOH + 8 H^{ + } + 8 e^{ - }$	−193.44	−96.72
$4 CH_{4} + H_{2} O \to CH_{3} CH_{2} CH_{2} CH_{2} OH + 8 H^{ + } + 8 e^{ - }$	−113.48	−28.37
$4 CH_{4} + H_{2} O \to CH_{3} CH(CH_{3} )CH_{2} OH + 8 H^{ + } + 8 e^{ - }$	−104.87	−26.22
$2 CH_{4} + H_{2} O \to CH_{3} CH_{2} OH + 4 H^{ + } + 4 e^{ - }$	−31.06	−15.53
$CH_{4} + H_{2} O \to CH_{3} OH + 2 H^{ + } + 2 e^{ - }$	+14.83	+14.83

Reduction half reactions	ΔG (kJ/mole oxidant)	ΔG (kJ/electron-pair)
$Fe^{3 + } + e^{ - } \to Fe^{2 + }$	−70.22	−140.44
$MnO_{2} + 4 H^{ + } + 2 e^{ - } \to Mn^{2 + } + 2 H_{2} O$	−77.65	−77.65
$NO_{3}^{ - } + 10 H^{ + } + 8 e^{ - } \to NH_{4}^{ + } + 3 H_{2} O$	−272.75	−68.15
$SO_{4}^{2 - } + 10 H^{ + } + 8 e^{ - } \to H_{2} S + 4 H_{2} O$	+178.14	+44.53

Standard transformed ΔGs were calculated at pH of 7, 25 °C, and an ionic strength of 0.25 M as described by Alberty [64]

**Fig. 3:**
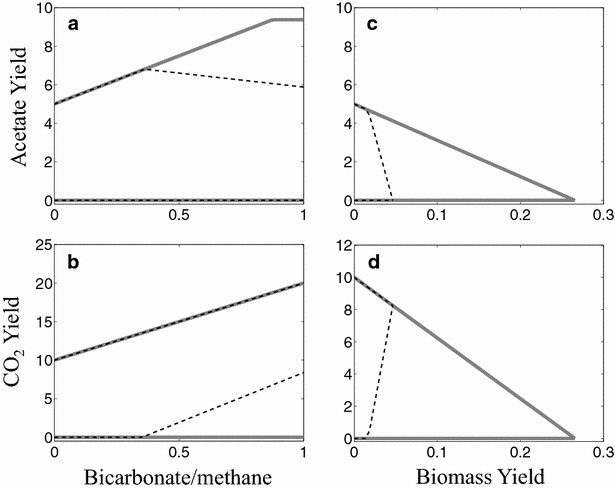
Acetate and carbon dioxide production yields as a function of bicarbonate to methane ratio (**a**, **b**) and biomass yield (**c**, **d**) using Fe^3+^ (*solid lines*) or SO_4_^2−^ (*dashed lines*) as external electron acceptors. Methane was the sole carbon source for generating the plots shown in *panels* (**c**) and (**d**). All yields are per 10 mmol of methane

The model predicts a maximum acetate production (0.5 mol/mol-methane), constrained only by stoichiometry for both Fe^3+^ and SO_4_^2−^ during growth on only methane. This yield is further increased to 0.94 mol/mol-methane at an optimal HCO_3_^−^/CH_4_ ratio of 0.88 for ferric-dependent methanotrophy, and 0.68 mol/mol-methane at an optimal HCO_3_^−^/CH_4_ ratio of 0.36 during sulfate-dependent methanotrophy (Fig. 3a). The improvement in acetate yield arises from the reduction in the fraction of methane oxidized via the methylotrophic pathway from 50 to 6 % and 32 % during ferric- and sulfate-dependent methanotrophy, respectively. However, a complete reversal of the aceticlastic pathway with a co-utilization ratio of one could not be achieved using either electron acceptor due to thermodynamic restrictions during sulfate-dependent methanotrophy and reduced ferredoxin availability during ferric-dependent methanotrophy. Under sulfate-dependent methanotrophy, the minimum essential flux through the methylotrophic pathway enables thermodynamic coupling with sulfate reduction for the generation of free energy equivalents. Mandatory channeling of electrons towards ferric ions by the electron bifurcation mechanism decreases available reduced ferredoxin for acetate synthesis during ferric-dependent methanotrophy. Despite the exergonic nature and the ATP generating capability of the acetate production pathway, it is never essential (minimum acetate production is always zero) at any HCO_3_^−^/CH_4_ ratio due to the fact that there exist other competing products and an electron acceptor-driven chemiosmotic ATP synthesis.

CO_2_ production remains non-essential during ferric-dependent methanotrophy as revealed by the model (Fig. 3b) due to the fact that reductive carboxylation of acetyl-CoA allows the production of many different thermodynamically feasible products. In contrast, CO_2_ production for sulfate-dependent methanotrophy beyond a HCO_3_^−^/CH_4_ ratio of 0.36 becomes mandatory. Beyond this ratio, increased CO_2_ production via the methylotrophic pathway serves to offset the free energy increase associated with uptake of bicarbonate. Carbon channeling towards the methylotrophic pathway leads to increased CO_2_ production thus decreasing methane flow towards other major products, thereby adversely affecting acetate and biomass yields at HCO_3_^−^/CH_4_ ratios beyond 0.36. The trade-off plot between the products of AOM and biomass did not reveal any thermodynamic restrictions in the solution space during ferric-dependent methanotrophy (Fig. 3c, d). However, the model predicts that acetate becomes thermodynamically constrained beyond a biomass yield of 0.018 for sulfate-dependent methanotrophy. Up to this yield value, the minimum required CO_2_ production remains zero due to the fact that either acetate or CO_2_ production pathways can generate the necessary free energy equivalents, ATP and reducing equivalents for biomass production. At biomass yields above 0.018, CO_2_ production becomes mandatory.

**Fig. 4:**
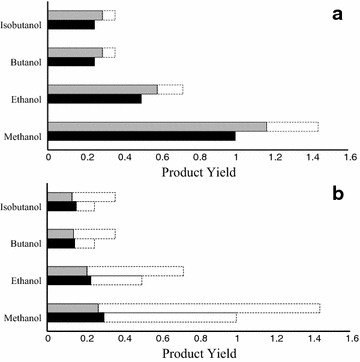
Biofuel yields using methane (*shaded in black*) as the sole carbon source and HCO_3_^−^/CH_4_ (*shaded in gray*) with a ratio of 0.44 for ferric-dependent (**a**) and sulfate-dependent (**b**) methanotrophy. The *dashed bars* denote the excess carbons that could not be incorporated into the product due to thermodynamic restrictions. Ferric-dependent methanotrophy predicted increased yields at higher HCO_3_^−^/CH_4_ ratios, but the carbon conversion was less than 100 %. Sulfate-dependent methanotrophy cannot achieve 100 % carbon conversion at any HCO_3_^−^/CH_4_ ratio due to mandatory co-production of thermodynamically feasible by-products. Product yields are in mol per mol methane

The production of ethanol, butanol, isobutanol, and methanol is thermodynamically feasible through both ferric-dependent and sulfate-dependent methanotrophy (Fig. 4). However, complete carbon conversion of methane to candidate biofuel molecules is thermodynamically feasible only for ferric-dependent methanotrophy with methane as the sole carbon source (Fig. 4a) due to the favorable thermodynamics of coupling the biofuel production pathways by ferric reduction (see Table 3). Upon co-utilization of methane and bicarbonate, electron bifurcation limits the availability of reduced ferredoxin for fixing CO_2_ by Cdh to produce acetyl-CoA (biofuel precursor), thereby restricting maximum achievable biofuel yield (Fig. 4a). Moreover, biofuel production pathways require additional energy in the form of NAD(P)H necessitating elevated amounts of reduced F_420_ at increasing bicarbonate to methane ratios which is also controlled by electron bifurcation. Incorporation of bicarbonate into methanol occurred via the CO_2_ reduction pathway (reversal of the methylotrophic pathway) as opposed to CO_2_ reduction by acetyl-CoA synthesis, causing all electrons to be generated by the ANME-MCR. During sulfate-dependent methanotrophy, none of the products could be produced with the complete carbon conversion efficiency due to the fact that coupling biofuel production with SO_4_^2−^ reduction remains thermodynamically infeasible (see Table 3) requiring the co-production of by-products such as acetate or CO_2_. As a consequence of this, co-utilization of bicarbonate and methane is not supported (see Fig. 4b). Although both oxidation of methane to methanol and reduction of sulfate to sulfide are thermodynamically infeasible, methanol can be still produced with SO_4_^2−^ due to coupling with the concomitant production of CO_2_. The lower bound for the production of all biofuel molecules is zero indicating that their production is not growth-coupled when methane is either the sole carbon source or co-utilized with bicarbonate.

### Interplay between electron acceptors and by-products of AOM at no growth

**Fig. 5:**
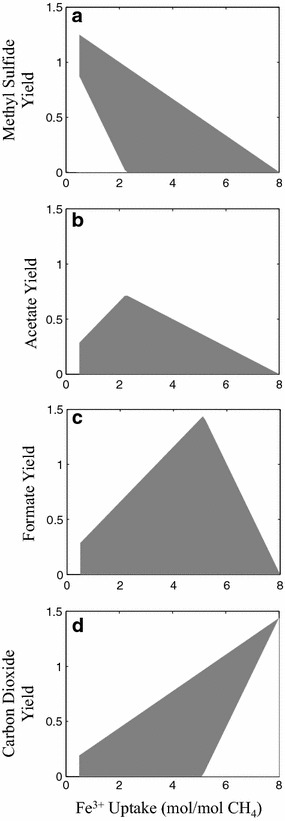
Thermodynamically feasible production envelope (*highlighted in grey*) of methyl sulfide (**a**), acetate (**b**), formate (**c**), and carbon dioxide (**d**) as a function of Fe^3+^ uptake predicted by the iMAC868 model under no growth. All product yields and Fe^3+^ uptake are defined as mol per mol methane

The interplay between the external electron acceptor choice and various products of AOM is pictorially illustrated (see Fig. 5) using feasible production envelopes for growth-arrested cells. We constrained the model for zero growth, ATP production for only maintenance requirements, and bicarbonate to methane ratio of 0.44. Analysis of the product profiles predicted by the iMAC868 model, based on the imposed constraints, identifies acetate as the main product of co-utilization of methane with bicarbonate along with the possible production of formate, CO_2_, and methyl sulfide (Fig. 5). A minimum Fe^3+^ uptake (i.e., 0.5 mol/mol-methane) is necessary to maintain thermodynamic feasibility of the observed solution spaces (Fig. 5a–d) at which methyl sulfide is found to be essential (Fig. 5a) due to the fact that it is the least oxidized by-product of methanotrophy by *M. acetivorans*. The maximum methyl sulfide yield at this Fe^3+^ uptake exceeds methane uptake indicating that bicarbonate is reduced via the methylotrophic pathway. The reversal of the methylotrophic pathway, however, is limited by the availability of reducing equivalents provided by Mcr, thereby resulting in an incomplete conversion of substrate carbons (methane and bicarbonate) to methyl sulfide. Increasing Fe^3+^ uptake allows more flux through the methylotrophic pathway, thereby generating additional intracellular CO_2_ for an increased acetate production of up to a maximum of 0.71 mol/mol-methane at an Fe^3+^ uptake of 2.2 mol/mol-methane (Fig. 5b). At this uptake rate, acetate can be produced as the sole product of methanotrophy resulting in non-essentiality of methyl sulfide production. Beyond this Fe^3+^ uptake rate, acetate production decreases due to the paucity of methyl-coenzyme M arising from increased flux through the methylotrophic pathway and channeling of electrons towards Fe^3+^ reduction via the membrane-bound electron transport chain. This increase in methylotrophic pathway flux also increases the yield of formate, an intermediate of this pathway. Maximum formate yield is found to be 1.44 mol/mol-methane at a Fe^3+^ uptake of 5.1 mol/mol-methane (Fig. 5c) where all taken up carbons are converted to formate. Beyond this Fe^3+^ uptake rate, CO_2_ production becomes essential so as to generate sufficient electrons for reduction of Fe^3+^ (Fig. 5d). A consequence of the essentiality of CO_2_ is the reduction of maximum formate yield. At a maximum Fe^3+^ uptake of 8 mol/mol-methane, only CO_2_ is produced due to the fact that it is the most oxidized form of carbon that can be produced by *M. acetivorans*.

## Conclusions

An updated genome-scale metabolic reconstruction (iMAC868) for the archaeon *Methanosarcina acetivorans* that integrates the latest literature findings and provides complete pathways and electron flow systems for reversing methanogenesis is introduced. Upon improving growth and gene-knockout outcome prediction for *M. acetivorans* grown on its native substrates, the model was used in a prospective mode for assessing thermodynamically feasible methanotrophic pathways leading to the production of biofuel candidate molecules such as methanol, ethanol, butanol, and isobutanol. We found that anaerobic methanotrophy favored the production of acetate and CO_2_ as they provide free energy equivalents to support growth. Co-utilization of CO_2_ (in the form of bicarbonate) and methane was feasible for certain ratios leading to improved carbon yields for acetate and biofuel molecules. Re-routing of a fraction of carbon towards CO_2_ was found to be a recurring mechanism for driving growth and production within thermodynamically constrained metabolic states. Finally, the availability in excess of inorganic electron acceptors resulted in a switch between substrate-level and chemiosmotic ATP synthesis. Thermodynamic constraints were often the limiting factor in product yields. Unsurprisingly, the inability to completely reverse the aceticlastic pathway in the absence of an external electron acceptor was confirmed.

The theoretical limits of external electron acceptor utilization possibilities to drive the reversal of methanogenesis were thoroughly explored. Key challenges that confound the fidelity of model predictions are still unknown sodium gradient requirements, substrate-dependent regulation and the detailed mechanism of electron transport from internal electron carriers to their external counterparts. Shedding light to these questions will require systematic experimental investigations to confirm or refute electron flow paths guided by the rapidly expanding modeling infrastructure.

## Methods

### Model assembly and growth/product formation predictions

The updated genome-scale metabolic model for *M. acetivorans*, iMAC868, was constructed by appending genes and reactions from iVS941 to iMB745. The stoichiometric coefficients of the ions associated with the membrane-bound electron transport chain were updated based on recent findings. This includes Rnf, ATP synthase and the Na^+^/H^+^ antiporter Mrp. All reactions in the model were checked for mass and charge balances and corrected if necessary. Five reactions from amino acid, two from tRNA, one from cofactor biosynthesis pathways, three from methanogenesis, and three metabolite transport reactions required elemental and charge rebalancing. The biomass equation formulation of iMB745 model was adopted in the new iMAC868 model as reported previously [36]. It was ensured that all biomass precursors could be independently produced in the model to avoid feasibiltiy tolerance related errors [60]. The iMAC868 model is available in Excel format in Additional file 1. All reaction fluxes are in mmol/gDCW-h except for the reaction representing cell biomass formation that is expressed in h^−1^. The medium composition was assumed to be a defined high-salt medium [29]. The model was assembled in a format compatible for flux balance analysis [61]. FBA optimization problems were solved by GNU Linear Programming Kit (GLPK) (http://www.gnu.org/software/glpk/) and Gurobi (http://www.gurobi.com) solvers in Matlab using COBRA toolbox [62]. Flux variability analysis (FVA) was performed to obtain range of fluxes under optimal growth conditions as described previously [63]. Both FBA and FVA problems incorporated overall thermodynamic feasibility constraints (overall ΔG ≤ 0). FBA was performed by solving the following Linear Programming (LP) problem:

$$\begin{array}{*{20}c} {Maximize } & {v_{biomass} } & {} & {} \\ {Subject \, to} & {\mathop \sum \limits_{j} S_{ij} v_{j} = 0,} & {\forall i \in I, j \in J} & {(1)} \\ {} & {\mathop \sum \limits_{j} \varDelta G_{j} v_{j} \le 0,} & {\forall j \in J^{ex} \mathop \cup \nolimits \, \{ biomass\} } & {(2)} \\ {} & {LB_{j} \le v_{j} \le UB_{j} ,} & {\forall j \in J\backslash \{ Ex - methane, \,ATPM\} } & {(3)} \\ {} & {v_{Ex - methane} = - 10} & {} & {(4)} \\ {} & {v_{ATPM} \ge 2.5} & {} & {(5)} \\ {} & {\varDelta G_{j} = \varDelta G_{j}^{met} , } & {\forall j \in J^{ex} \mathop \cup \nolimits \, \{ biomass\} } & {(6)} \\ {} & {v_{j} \in {\mathbb{R}}} & {\forall j \in J} & {} \\ \end{array}$$

where sets, variables, and parameters are defined as follows:

Sets:

- *I* = {*i*|*i* = 1,2,…,*M*} = Set of metabolites in the stoichiometric model
- *J* = {*j*|*j* = 1,2,…,*N*} = Set of reactions in the stoichiometric model
- *J*^*ex*^ = {*j*|*j* = 1,2,…,*N*^*ex*^} = Set of exchange reactions in the stoichiometric model

Variables:

- *v*_*j*_ = Flux of reaction $j \in J$
- *v*_*biomass*_ = Flux of the biomass formation reaction

Parameters:

- *S*_*ij*_ = Stoichiometric coefficient of metabolite $i \in I$ in reaction $j \in J$
- *UB*_*j*_ = Upper bound for the flux of reaction $j \in J$
- *LB*_*j*_ = Lower bound for the flux of reaction $j \in J$
- $v_{Ex - methane}$ = Flux of the methane exchange reaction
- $v_{ATPM}$ = Flux of the non-growth associated maintenance ATP reaction
- $\varDelta G_{j}^{met}$ = The $\varDelta G$ of formation of the metabolite associated with exchange reaction $j \in J^{ex} \mathop \cup \nolimits \{ biomass\}$

All standard transformed ΔG values were calculated at pH of 7, temperature of 25 °C and ionic concentration of 0.25 M [64] listed in Additional file 1. The upper bound of the free energy of biomass formation (ΔG_biomass_) is estimated to be 3750 J/gDCW. This value is identified so as the overall stoichiometries for growth on acetate and methanol (see below) remain thermodynamically feasible:

$$10 \,CH_{3} COOH \to 9.3\, CH_{4} + 9.3 \,CO_{2} + 0.026\,\, biomass$$

$$10 \,CH_{3} OH \to 6.4 \,CH_{4} + 1.5\, CO_{2} + 6 \,H_{2} O + 0.052 \,\,biomass$$

The iMAC868 model arrived at these overall conversion stoichiometries by solving the FBA problem subject to constraints (1), (3), and (5) only.

In the above LP problem, the flux of the biomass is maximized subject to the constraints of stoichiometry (1), thermodynamics (2), metabolic network fluxes (3), fixed uptake of methane (4), minimum requirements of maintenance ATP of 2.5 mmol/gDCW-h (5), and fixed ΔG values of input/output metabolites to/from the system (6). Flux ranges for target products were obtained by iteratively solving the above LP problem to minimize and maximize all *v*_*j*_ separately subject to stoichiometric and thermodynamic constraints.

### Formulation of R-GPR to integrate ‘-omics’ data into the metabolic model

Gene-protein-reactions (GPRs) associations in the iMAC868 model were thoroughly assessed and 64 GPRs were corrected using a list of 781 newly revised gene annotations (Additional file 2) along with database entries from KEGG [65], MetaCyc [66], BRENDA [67], and TransportDB [68]. Following this, R-GPR switches were implemented using a dataset of quantitative protein levels for over 250 genes of *M. acetivorans* grown with acetate and methanol [34]. This dataset is given in Additional file 1. The R-GPR approach allows for the incorporation of ‘omics’ data for conditional switching on/off of reactions allowing improved gene-knockout predictions by providing an insight into the likelihood of a reaction to be active or inactive under specific growth conditions. The following systematic procedure elaborates this conditional activation/inactivation of reactions by the R-GPR switches:

Step 1: Calculate the ratio of protein abundance under different growth substrates. For each gene *k* in the total gene set *K*, the value *c*_*k*_ was calculated as the ratio of protein abundance for cells grown on substrate 1 to cells grown on substrate 2 where substrate 1 and 2 can be any of acetate or methanol.
Step 2: Compare the ratio *c*_*k*_ with a pre-defined cutoff value (i.e., equal to 25 %). If the ratio *c*_*k*_ is below or equal to the cutoff value then gene *k* is added to a candidate list *G* for which the feasibility of removing the corresponding reactions is evaluated.
Step 3: Re-evaluate GPRs within list *G*. The GPR for each reaction is re-evaluated assuming that all genes in list *G* are eliminated. If re-evaluation of the GPR reveals no associated gene then the reaction is added to the set *J*^*exp*^ that contains the candidate reactions for removal.
Step 4: The following mathematical formulation is used to identify the maximum number of reactions in the set *J*^*exp*^ that can be removed from the model without dropping the biomass yield below the experimental value:

$$\begin{array}{*{20}c} {Minimize} & {\mathop \sum \limits_{{j \in J^{exp} }} y_{j}} & {} & {} \\ {Subject \, to} & {\mathop \sum \limits_{j} S_{ij} v_{j} = 0,} & {\forall i \in I, j \in J} & {(7)} \\ {} & {y_{j} LB_{j} \le v_{j} \le y_{j} UB_{j} ,} & {\forall j \in J^{exp} \backslash \left( {J^{on} \mathop \cup \nolimits J^{off} } \right)} & {(8)} \\ \begin{aligned} \hfill \\ \hfill \\ \hfill \\ \hfill \\ \end{aligned} & \begin{aligned} LB_{j} \le v_{j} \le UB_{j} , \hfill \\ v_{j} = 0 \hfill \\ v_{biomass} \ge v_{biomass,exp} \hfill \\ v_{j} \in {\mathbb{R}},\text{ }y_{j} \in \{ 0,1\} \hfill \\ \end{aligned} & \begin{aligned} \forall j \in \left( {J\backslash J^{exp} } \right)\mathop \cup \nolimits J^{on} \hfill \\ \forall j \in J^{off} \hfill \\ \hfill \\ \forall j \in J \hfill \\ \end{aligned} & \begin{aligned} (9) \hfill \\ (10) \hfill \\ (11) \hfill \\ \hfill \\ \hfill \\ \end{aligned} \\ \end{array}$$

where sets, variables, and parameters in this MILP problem have the same definition as those defined earlier for the LP problem except for the followings:

Sets:

- *J*^*exp*^ = {*j*|*j* = 1, 2, …, *N*^*exp*^} = Set of reactions whose GPRs are evaluated due to availability of experimental data (i.e., proteomic data)
- *J*^*on*^ = {*j*|*j* = 1, 2, …, *N*^*on*^} = Set of reactions for which the evaluation of their GPR indicates active reactions
- *J*^*off*^ = {*j*|*j* = 1, 2, …, *N*^*off*^} = Set of reactions for which the evaluation of their GPR indicates inactive reactions

Variables:

- *v*_*biomass*_ = Flux of the reaction producing biomass
- *y*_*j*_ = Binary variable associated with flux of *v*_*j*_

Parameters:

- *v*_*biomass,exp*_ = The experimentally measured biomass yield for the given substrate

Here, the sum of binary variables *y*_*j*_ is minimized subject to the limitations on stoichiometry (7), the flux of the reactions for which proteomic data is available but their GPRs are not evaluated because *c*_*k*_ is greater than the cutoff value (8), the flux of all other metabolic network reactions which do not belong to set *J*^*exp*^ along with those belong to set *J*^*on*^ (9), and the flux reactions which belong to set *J*^*off*^ (10), and the flux of the reaction producing biomass being equal or greater than the experimentally measured biomass yield for the given substrate (11).

### Representation of external electron acceptor in the metabolic network of iMAC868

Electron acceptor reactions are modeled using “electron acceptor equivalents (EAE)”, which serves to drain electron pairs from the model. Each electron pair is drained from reduced methanophenazine. The general form of the electron acceptor reaction used in this model is:

$$Reduced\,\,methanophenazine \,+\, aH^{ + } [c] \to Oxidized \,\,methanophenazine \,+\, EAE \,+\, bH^{ + } [e]$$

here, a and b correspond to the excess protons drained from the cytosol and secreted into the extracellular medium, respectively. These values are electron acceptor specific and can be obtained from the corresponding electron acceptor reduction reactions described in Table 3. In order to make EAE a balanced metabolite, an exchange reaction describing the draining of EAE is added to the model, with ΔG_EAE_ corresponding to the ΔG_red_ (kJ/electron-pair) of the specific electron acceptor described in Table 3.

### Optimization of Na^+^/H^+^ ratios of ATP synthase and Mrp antiporter

To find the optimal Na^+^/H^+^ stoichiometric ratios of ATP synthase and Mrp matching best the observed growth yields, Flux Balance Analysis (FBA) was used as described earlier in this section. FBA optimization problems were solved at varying Na^+^/H^+^ ratios of 3:1, 1:1, 1:3, 2:1, and 1:2 for ATP synthase and at varying Na^+^/H^+^ ratios of 3:1, 2:1, 1:1, 1:2, and 1:3 for Mrp. These ratios were selected based on recent findings involving archaeal ATP synthase and their dependence on Mrp. Welte and Deppenmeier [42] estimated that 3–4 translocated ions (H^+^ or Na^+^) are required to synthesize one molecule of ATP based on the measured electrochemical ion gradients available for a limited number of methanogens. Jasso-Chavez et al. [40] proposed that Mrp is used for optimization of the thermodynamic efficiency of the ATP synthase in *M. acetivorans*. The objective function was the maximization of cellular growth. This procedure was repeated for both acetate and methanol as sole carbon sources. The sum squared error (SSE) between the predicted and observed growth yields were calculated and results are shown in Table 4.

**Table 4:** SSE between predicted (by iMAC868) and observed growth yields for acetate- and methanol-grown cells at different ratios of Na^+^ and H^+^ exchange by Mrp antiporter and varying ratios of Na^+^ and H^+^ uptake by ATP synthase

Substrate	Ratio of Na^+^ to H^+^ exchange by Mrp antiporter	Ratio of Na^+^ to H^+^ uptake by ATP synthase				
		3:1	1:1	1:3	2:1	1:2
Methanol	3:1	2.11E−04	7.75E−06	2.21E−04	1.75E−02	2.53E−05
	2:1	***1.40E−07***	8.58E−05	2.86E−04	1.67E−04	*1.40E−07*
	1:1	4.57E−04	4.57E−04	4.57E−04	8.33E−05	8.33E−05
	1:2	1.39E−03	6.73E−04	1.43E−04	1.43E−04	3.32E−05
	1:3	1.57E−03	5.44E−04	1.26E−06	1.73E−04	1.78E−04
Acetate	3:1	7.97E−06	2.13E−04	Infeasible	3.93E−02	4.76E−05
	2:1	***8.00E−06***	7.34E−05	2.96E−04	3.44E−04	*8.00E−06*
	1:1	8.05E−06	8.05E−06	8.05E−06	5.51E−04	5.51E−04
	1:2	8.09E−06	1.82E−04	7.45E−04	7.45E−04	2.10E−03
	1:3	8.10E−06	3.45E−04	1.74E−03	8.36E−04	3.34E−03

Only the 2:1 ratio of Na^+^ and H^+^ for Mrp satisfies the closest match between predicted and observed growth yields for both substrates. ATP synthase can uptake either 3 Na^+^ and 1 H^+^ (bold italics) or 1 Na^+^ and 2 H^+^ (italics), which are rendered equivalent by Mrp. Infeasibility arises from the inability to meet minimum ATP requirements

## Additional files

10.1186/s12934-015-0404-4 iMAC868 metabolic reconstruction.
10.1186/s12934-015-0404-4 *M. acetivorans* gene re-annotation list.

## Abbreviations

ANME: anaerobic methanotrophic archaea
AOM: anaerobic oxidation of methane
GSM: genome-scale metabolic model
iMAC868: in silico *Methanosarcina acetivorans* metabolic model containing 868 genes
GPR: gene-protein-reaction associations
R-GPR: regulatory gene-protein-reaction associations
EAE: electron acceptor equivalent
